# Clinical significance of HPV DNA and EGFR mutations in Egyptian NSCLC

**DOI:** 10.1186/s13027-025-00712-9

**Published:** 2025-12-01

**Authors:** Shaimaa Saeed Mohamed, Zeinab K. Hassan, Ahmed M. Fahmy, Amira Salah El-Din Youssef, Nasra F. Abdel Fattah, Amira I. Khater, Hany K. Soliman, Mohamed M. Hafez

**Affiliations:** 1 Virology and Immunology Unit, Cancer Biology Department, National Cancer Institute, Cairo University, Cairo, Egypt; 2 Surgical Pathology Department, National Cancer Institute, Cairo University, Cairo, Egypt; 3 Cancer Epidemiology & Biostatistics Department, National Cancer Institute, Cairo University, Cairo, Egypt; 4 Virology and Immunology Unit, Cancer Biology Department, National Cancer Institute, Fom El-Khalig, Cairo, 11796 Egypt

**Keywords:** NSCLC, EGFR mutations, HPV-DNA, EBV-DNA, Adenocarcinoma, Squamous cell carcinoma, Overall survival, Molecular profiling, Viral oncogenesis

## Abstract

**Background:**

Non-small cell lung cancer (NSCLC) remains a leading cause of cancer-related mortality worldwide. While molecular profiling particularly EGFR mutation analysis has transformed therapeutic approaches, the role of viral oncogenesis, such as human papillomavirus (HPV) infection and Epstein-Barr virus (EBV), in NSCLC is less well understood. In this study, we investigated the clinical relevance of HPV/EBV infection and EGFR mutations in NSCLC.

**Methods:**

This retrospective study analyzed tumor tissues from 93 patients with NSCLC, as well as lung tissues from 10 normal adjacent samples. HPV-DNA detection and genotyping were performed using PCR-based methods, alongside EBV-DNA analysis. EGFR mutations were assessed via exon-specific PCR amplification. Associations between HPV/EGFR status and clinicopathological parameters were evaluated, and Kaplan–Meier survival analysis was used to assess overall survival (OS) according to molecular profiles.

**Results:**

HPV-DNA was detected in 31.2% of NSCLC tumors, while EBV-DNA was identified in 2.15%, with no positivity in normal adjacent tissues. HPV 16 was the most prevalent genotype, especially among adenocarcinoma (AC) cases. EGFR mutations were found in 20.4% of patients, predominantly in AC, with exon 19 deletions and L858R mutations being the most common. While HPV status showed no significant correlation with most clinical features, a strong inverse association was observed with surgical operability (*p* < 0.001). Survival analyses revealed that HPV-positive patients had shorter OS compared to HPV-negative counterparts. Patients with EGFR mutations exhibited longer OS, particularly in the HPV-negative subgroup, while the HPV-positive/EGFR-wild type group had the poorest outcomes.

**Conclusion:**

This study demonstrates distinct prevalence patterns and prognostic implications of HPV infection and EGFR mutations in NSCLC. EGFR-mutant status correlated with improved survival, while HPV positivity was associated with inoperable disease and poorer outcomes. Further validation in larger cohorts is needed before clinical application.

**Clinical trial number:**

Not applicable.

## Introduction

Lung cancer (LC) remains the leading cause of cancer-related mortality worldwide, with an estimated 2.5 million new cases and 1.8 million deaths reported in 2022. In Egypt, LC ranks fifth among all malignancies, accounting for 7,643 new cases and 6,805 deaths [1]. Non-small cell lung cancer (NSCLC) constitutes approximately 85% of LC cases globally, and about 40% of LC patients in Egypt are diagnosed with this subtype [2]. Although tobacco smoking is the principal risk factor for LC, its pathogenesis involves a multifactorial interplay of genetic, environmental, and infectious determinants [3]. Notably, 10–15% of LC cases occur in never-smokers, among whom LC ranks as the seventh leading cause of cancer-related mortality [3, 4].

Infectious agents are implicated in nearly 20% of all human malignancies, with viruses accounting for approximately 15% [5]. Viral oncogenesis typically involves integration of viral DNA into the host genome, leading to dysregulation of proto-oncogenes or inactivation of tumor suppressor pathways [3]. Among oncogenic viruses, human papillomavirus (HPV) and Epstein–Barr virus (EBV) have been proposed as potential cofactors in lung carcinogenesis; however, their exact roles remain controversial [6].

HPV is a small, circular, double-stranded DNA virus known to cause a wide spectrum of benign and malignant lesions. Globally, HPV is responsible for nearly 29.5% of all viral infections and contributes to approximately 4.5% of newly diagnosed cancers [6]. To date, nearly 400 HPV genotypes have been identified and classified based on oncogenic potential into high-risk types (e.g., 16, 18, 31, 33, and 45) and low-risk types (e.g., 6, 11, 42, 43, and 44). In addition, approximately 200 animal HPV types have been recognized [7]. Since Syrjänen first proposed a link between HPV and lung carcinogenesis in 1979 [8–13], numerous studies have investigated this association. The first meta-analysis, which included 7,381 LC cases, reported HPV DNA detection in 22.4% of samples [11]. Nonetheless, the contribution of HPV to LC, particularly NSCLC, remains inconclusive due to marked geographic and methodological variability across studies.

EBV, a lymphotropic double-stranded DNA virus, was the first human tumor virus identified and is known to infect more than 90% of adults worldwide. It is estimated to account for approximately 1.5% of all cancers and has been detected in both squamous cell carcinoma (SCC) and lung adenocarcinoma (LA) [3, 14]. A recent meta-analysis (2023) reported that EBV infection increases the risk of LC more than fourfold, with a prevalence of 34.8% in NSCLC and 21.2% in small-cell lung cancer (SCLC) [14]. However, data on the prevalence of HPV and EBV infections in Egyptian NSCLC patients remain scarce.

Genetic alterations also play a crucial role in LC development, particularly among nonsmokers. Somatic mutations in key driver genes such as epidermal growth factor receptor (EGFR), tumor protein 53 (TP53), and Kirsten rat sarcoma viral oncogene (KRAS) are frequently implicated in pathways regulating proliferation, differentiation, and apoptosis [15–17]. A study in a Croatian cohort demonstrated that EGFR-mutated lung adenocarcinomas exhibited higher rates of HPV and EBV infections, suggesting a possible viral contribution to their pathogenesis [3]. Similarly, a study from China reported that advanced LA patients harboring both HPV infection and EGFR mutations showed improved responses to EGFR tyrosine kinase inhibitors or platinum-based chemotherapy [4]. Nevertheless, the relationship between viral infection (HPV, EBV) and EGFR mutations in NSCLC remains poorly defined.

Although few studies in Egypt have explored EGFR mutation patterns in NSCLC [2, 18], none have examined the prevalence of HPV and EBV infections or their potential association with EGFR status in Egyptian patients. Given the paucity of data from Egypt, this study aimed to determine the prevalence of HPV and EBV infections in NSCLC, assess their association with EGFR mutations and clinicopathologic features, and explore their combined prognostic implications.

## Subjects and methods

### Study setting

A retrospective *cross-sectional study* was conducted on 93 Formalin-Fixed Paraffin-Embedded (FFPE) tissue samples obtained from the cancer pathology department, National Cancer Institute (NCI), from NSCLC patients, specifically AC and SCC, who were referred to the NCI, Cairo University, between March 2017 and September 2020. Additionally, 10 FFPE samples of normal adjacent tissue were collected to demonstrate the feasibility of detecting viral DNA in non-cancerous individuals. Ethical approval for our study (approval no. **CB2401-102-046**) was granted by the Institutional Review Board of the Egyptian NCI, Cairo University, in accordance with ICH-GCP guidelines. NSCLC patients who had not received any form of treatment such as chemotherapy, radiotherapy, tyrosine kinase inhibitors, or immunotherapy prior to undergoing biopsy or lobectomy were included in this study.

### Collection of patients’ data

The data of the patients were obtained from their medical records maintained in the biostatistics and epidemiology department at NCI, Cairo University. The extracted data encompassed the following variables: age at diagnosis, sex, smoking history, presence of metastasis, histologic grade, laterality, histologic type, treatment data after biopsy or lobectomy, and the most recent follow-up date.

### Sample preparation and DNA extraction

The FFPE tissue blocks were sectioned into eight slices (each of the eight sections is 5–10 micron thick). A pathologist reviewed and examined a representative hematoxylin and eosin (H&E)-stained slide for each sample to confirm that the sections contained a sufficient percentage of representative tumor cells. Genomic and viral DNA were extracted from the selected FFPE tissue sections of NSCLC/AC and NSCLC/SCC patients, as well as normal adjacent tissue, using the QIAamp DNA FFPE Tissue Kit (Qiagen, Germany) (catalog number: 51104) following the manufacturer’s instructions. The extracted and purified DNA was then stored at -20 °C until it was required.

### Molecular detection of viral DNAs

#### Detection of HPV and its genotypes (16, 18, 33, 58)

DNA samples from all patients and normal adjacent tissue were analyzed using a qualitative conventional PCR technique to detect the HPV Late 1 (L1) region, employing common primers MY09 and MY11. PCR amplification was carried out in a final reaction volume of 25µL, consisting of 1X AmpliTaq PCR master mix (Applied Biosystems, Barcelona, Spain), 300nM of each primer, and nuclease-free water to complete the volume. Amplification was performed using a Veriti 96-well thermal cycler (Cat. No. 4375305, Thermo Fisher Scientific Inc., USA).

To ensure the accuracy and reliability of the results, both positive and negative controls were included in each PCR assay. MCF7 and HeLa cells were used as positive controls for HPV type 16 and HPV type 18, respectively. The amplified PCR products were analyzed by electrophoresis, with 15 µL of each product loaded onto a 2% agarose gel (Sigma) prepared in 1X Tris-acetate-EDTA (TAE) buffer. The gel was stained with 0.5 µg/mL ethidium bromide, and bands were visualized under ultraviolet (UV) transillumination, followed by photographic documentation. A 100 bp DNA ladder (Genedirex, Taiwan) was used as a molecular weight marker to compare product sizes. The specificity of qualitative PCR for detecting HPV DNA was assessed by testing samples that were positive for several viruses, including Herpes Simplex Virus 1 (HSV1), Herpes Simplex Virus 2 (HSV2), Adenovirus (Adv.), HMTV, HPV, Cytomegalovirus (CMV), and EBV. A qualitative PCR assay was conducted following the established protocols from prior publications.

The positive samples for HPV were analyzed to identify genotypes, specifically16,18, 33, and 58, utilizing a real-time detection system (Applied Biosystems^®^7500 Fast Real-Time PCR System Thermal Cycling Block, USA) according to the established protocol.

Amplification was performed in a total reaction volume of 25 µL using DiaStar™ OneStep Multiplex qRT-PCR mix (Techno 5-ro, Korea). The reaction mixture included 300 nM of each forward and reverse primer and corresponding probe for HPV genotypes 16 (Vi07921925_s1), 18 (Vi07921926_s1), 33 (Vi07921969_s1), and 58 (Vi07922389_s1) (Applied biosystem, USA), along with 100 ng of extracted DNA from samples and HPV-positive samples.

Thermal cycling conditions were as follows: an initial denaturation at 95 °C for 5 min, followed by 40 cycles of denaturation at 95 °C for 45 s, annealing/extension at 60 °C for 1 min. Following amplification, the real-time PCR data were analyzed using the accompanying software provided by the qPCR instrument (Applied Biosystems ^®^7500 Fast Real-Time PCR Software). Cycle threshold (Ct) values were automatically calculated for each sample. A sample was considered positive for a specific HPV genotype if the Ct value was below the threshold of 35 cycles. Negative controls (no-template) were monitored to ensure the absence of contamination, while positive controls confirmed the reliability and specificity of the assay. Genotyping results were interpreted by comparing Ct values corresponding to each specific probe, and only samples showing amplification with genotype-specific primers and probes were reported as positive for that HPV type.

### Molecular detection of EBV

Epstein–Barr virus (EBV) detection was performed using a real-time PCR system (Applied Biosystems StepOne™ Real-Time PCR System, USA). The Artus EBV TM PCR Kit (Qiagen, Germany; Catalog No. 4501163), which targets the EBNA-1 gene, was used following the manufacturer’s instructions. PCR amplification was carried out in a total reaction volume of 25 µL, consisting of 15 µL of EBV TM master mix (containing primers and probe), 1 µL of internal control, and 10 µL of purified DNA (approximately 100 ng) from each sample. Thermal cycling conditions were as follows: an initial denaturation at 95 °C for 5 min, followed by 40 cycles of denaturation at 95 °C for 45 s, annealing/extension at 60 °C for 1 min. Following amplification, the real-time PCR data were analyzed using the accompanying software provided by the qPCR instrument (Applied Biosystems StepOne™ Software).

### EGFR mutational analysis

EGFR mutations were detected using the Amoy Dx EGFR 29 Mutations Detection Kit (Catalogue number: 8.01.20201 × 024 F, AmoyDx^®^, China), following the manufacturer’s instructions. The analysis was performed on an Applied Biosystems 7500 Fast Real-Time PCR equipment, USA Singapore). This kit utilizes Amplification Refractory Mutation System (ARMS) technology and Scorpion techniques, which utilize specific primers and fluorescent probes for real-time PCR detection of mutations. It detects 29 somatic mutations in the tyrosine kinase (TK) domain of EGFR gene including point mutations G719X (exon 18), T790M and S768I mutations (exon 20), and L858R, L861Q mutations (exon 21), 3 insertions in exon 20, 19 deletions in exon 19. The detected mutations are presented in Table 1.

**Table 1:** List of detected mutations and their corresponding catalogue of somatic mutation in cancer (COSMIC) IDs

Mutation	Exon	Base change	COSMIC ID
T790M	20	2369 C > T	6240
L858R	21	2573T > G	6224
L861Q	21	2582T > A	6213
S768I	20	2303G > T	6241
G719A	18	2156G > C	6239
G719S	18	2155G > A	6252
G719C	18	2155G > T	6253
Insertions	20	2307_2308ins9	12,376
Insertions	20	2319_2320insCAC	12,377
Insertions	20	2310_2311insGGT	12,378
Deletion	19	2235_2249del15	6223
		2235_2252 > AAT(complex)	13,551
		2236_2253del18	12,728
		2237_2251del15	12,678
		2237_2254del18	12,367
		2237_2255 > T (complex)	12,384
		2236_2250del15	6225
		2238_2255del18	6220
		2238_2248 > GC (complex)	12,422
		2238_2252 > GCA(complex)	12,419
		2239_2247del9	6218
		2239_2253del15	6254
		2239_2256del18	6255
		2239_2248TTAAGAGAAGC (complex)	12,382
		2239_2258 > CA (complex)	12,387
		2240_2251del12	6210
		2240_2257del18	12,370
		2240_2254del15	12,369
		2239_2251 > C (complex)	12,383

The ∆Ct was calculated for mutational status of each sample using the formula (∆Ct value = mutant FAM Ct value – external control FAM Ct value). The mutations were identified when the FAM mutant exhibited true amplification, and the ∆Ct value was below the ∆Ct cut-off.

### Statistical methodology

Data management and analysis were performed using Statistical Package for Social Sciences (SPSS) vs. 27. Numerical data were summarized using means and standard deviations or medians and ranges, as appropriate. Categorical data were summarized as numbers and percentages. Chi square or Fisher’s tests were used to compare between groups with categorical data, as appropriate. Kaplan–Meier method was used to estimate overall survival (OS). Patients alive at last follow-up were censored. Predictor and prognostic variables were related to survival using log rank test. To evaluate the impact of variables on the risk of death and to identify the independent prognostic ones affecting OS; univariate followed by multivariate Cox proportional hazard analyses were done. Multivariate analysis included variables significant in univariate one. All tests were two-sided. P-values < 0.05 were considered significant.

## Results

### EGFR mutation spectrum and molecular characterization

The data presented in Table 1 underscore the molecular diversity of EGFR mutations in NSCLC and reinforce the clinical importance of comprehensive genotyping to inform precision oncology approaches. Table 1 summarizes the genetic heterogeneity of EGFR mutations identified in NSCLC patients, highlighting their exon locations, nucleotide changes, and associated COSMIC (Catalogue of Somatic Mutations in Cancer) IDs. Among the most clinically significant mutations are: T790M (Exon 20, 2369 C > T, COSMIC ID: 6240), a well-known resistance mutation that emerges during treatment with first- or second-generation EGFR tyrosine kinase inhibitors (TKIs); L858R (Exon 21, 2573T > G, COSMIC ID: 6224), one of the most common sensitizing mutations conferring high responsiveness to TKIs; L861Q (Exon 21, 2582T > A, COSMIC ID: 6213), also associated with TKI sensitivity; S768I (Exon 20, 2303G > T, COSMIC ID: 6241), which has been linked to intrinsic or acquired resistance.

Activating mutations in Exon 18, including G719A (2156G > C, COSMIC ID: 6239), G719S (2155G > A, COSMIC ID: 6252), and G719C (2155G > T, COSMIC ID: 6253), are less frequent but clinically relevant, as they may respond to certain second-generation EGFR inhibitors. Exon 20 insertions, such as: 2307_2308ins9 (COSMIC ID: 12376), 2319_2320insCAC (COSMIC ID: 12377), and 2310_2311insGGT (COSMIC ID: 12378), are typically associated with resistance to standard TKIs, underscoring the need for alternative therapeutic strategies in patients harboring these variants. Exon 19 deletions represented the most diverse category, encompassing both classic and complex variants. These include: 2235_2249del15 (COSMIC ID: 6223), 2236_2250del15 (COSMIC ID: 6225), 2235_2252 > AAT (COSMIC ID: 13551), and 2239_2248TTAAGAGAAGC (COSMIC ID: 12382).

### Clinico-pathological findings

The clinical and pathological characteristics of the 93 patients diagnosed with NSCLC are summarized in Table 2. The mean age at diagnosis was 59.1 ± 9.6 years (range: 29–88), with a notable male predominance (68.8%; *n* = 64) compared to females (31.2%; *n* = 29). Regarding smoking status, 64.5% (*n* = 60) of patients were smokers including one female while 35.5% (*n* = 33) were non-smokers. In terms of histological subtype, the cohort was almost evenly divided between AC (50.5%; *n* = 47) and SCC (49.5%; *n* = 46). Tumor laterality was evaluated in 77 patients, with right lung involvement more commonly observed (59.7%; *n* = 46) than left-sided tumors (40.3%; *n* = 31).

**Table 2:** Clinical and pathological characteristics of non-small cell lung cancer (NSCLC) patients (*n* = 93)

Characteristics		Number	Percent
Age (years) *		59.1 ± 9.6	
Gender	Male	64	68.8
	Female	29	31.2
Smoking status	Non-smoking	33	35.5
	Smoking	60	64.5
Laterality (*n* = 77)	Left	31	40.3
	Right	46	59.7
Histopathologic type	Adenocarcinoma	47	50.5
	Squamous cell carcinoma	46	49.5
Stage (*n* = 90)	I	1	1.1
	II	14	15.6
	III	36	40.0
	IV	39	43.3
Grade	1	3	3.2
	2	55	59.1
	3	35	37.6
Surgery	No	59	63.4
	Yes	34	36.6
Type of treatment	CTH only	69	74.2
	CTH &RTH	18	19.4
	RTH only	6	6.5
Metastatic disease	No	22	23.7
	Yes	71	76.3

CTH: Chemotherapy, RTH: Radiotherapy. * Variable is presented as mean ± standard deviation

Clinical stage data were available for 90 patients. The majority presented with advanced-stage disease: Stage III (40.0%; *n* = 36) and Stage IV (43.3%; *n* = 39). Early-stage tumors were less frequent, with Stage II accounting for 15.6% (*n* = 14) and only one case (1.1%) diagnosed at Stage I. Regarding tumor differentiation, Grade 2 tumors were most common (59.1%; *n* = 55), followed by Grade 3 in 37.6% (*n* = 35), and Grade 1 in just 3.2% (*n* = 3).

Treatment modalities varied across the cohort. The majority of patients (74.2%; *n* = 69) received chemotherapy alone, while 19.4% (*n* = 18) underwent combined chemotherapy and radiotherapy, and 6.5% (*n* = 6) received radiotherapy only. Surgical interventions were performed in 36.6% (*n* = 34) of cases, whereas 63.4% (*n* = 59) did not undergo surgery, likely reflecting their advanced disease at presentation. Notably, 76.3% (*n* = 71) of patients exhibited metastatic disease at the time of diagnosis.

### Presence of viral DNA (HPV and EBV) in NSCLC patients and normal adjacent tissue

HPV-DNA was detected in **29 of 93 NSCLC cases (31.2%)**, while none of the 10 normal adjacent tissues tested positive, as shown in Table 3. Among the histological subtypes, **HPV was more frequently detected in SCC cases (39.1%**,** 18/46)** than in AC cases (23.4%, 11/47).

Among the 29 HPV-positive tumors, **HPV-16** was the most prevalent, identified in **5 cases (17.2%)**. Interestingly, HPV 16 was disproportionately found in HPV-positive **AC cases (36.4%)** compared to **SCC cases (5.5%)**, indicating histology-specific tropism or differing patterns of host-pathogen interaction. **HPV-18** was detected in only **one case (3.4%)**, while **HPV-33 and HPV-58** were not found in any samples (Table 4). Overall, **HPV-16** emerged as the only substantially represented high-risk genotype, with a notably higher frequency in AC compared to SCC.

As regards EBV DNA (*EBNA1* gene), it was detected in only 2.15% (*n* = 2) of NSCLC tissues while it was absent in normal adjacent tissue, as shown in Table 3. Both EBV-positive cases were exclusively recognized as poorly differentiated SCC, with one case exhibiting co-infection with HPV. Of note, both patients were smokers and were diagnosed at a metastatic stage.

**Table 3:** Presence of HPV, and EBV DNAs in FFPE tissue of NSCLC patients and normal adjacent tissues

Group	*N* = 93	HPV		EBV	
		Positive	Negative	Positive	Negative
NSCLC	N %	29 (31.2%)	64 (68.8%)	2 (2.15%)	91 (97.8%)
Normal adjacent tissues	*N* = 10 N %	0 (0.0%)	10 (100%)	0 (0.0%)	10 (100%)

**Table 4:** Distribution of HPV genotypes in HPV-Positive NSCL patients (*n* = 29)

Genotype	Positive (%)	Negative (%)
HPV-16	17.2	81.8
HPV-18	3.4	96.6
HPV-33	0.0	100
HPV-58	0.0	100

### HPV presence and its association with clinico-pathological features in NSCLC

As shown in Table 5, HPV DNA positivity in NSCLC patients did not significantly correlate with most clinico-pathological features, including age, gender, smoking status, histopathological subtype, tumor stage, grade, or presence of metastasis (*p* > 0.05 for all). Although not statistically significant, several trends were observed: HPV was more frequently detected in patients over 59 years of age (38.6%) compared to those 59 or younger (24.5%) (*p* = 0.141), in smokers (33.3%) versus non-smokers (27.3%) (*p* = 0.546), and in advanced-stage disease (33.3% in stage III–IV vs. 20.0% in stage I–II; *p* = 0.309). A HPV prevalence was higher in SCC (39.1%) than in AC (23.4%); however, the difference was not statistical significance (*p* = 0.102). A trend was observed between HPV and histopathological type (*p* = 0.102), though it did not reach statistical significance. Notably, a **highly significant association** was found between HPV status and surgical intervention (*p* < 0.001). HPV DNA was detected in only 5.9% of patients who underwent surgical resection, compared to 45.8% of those who did not undergo surgery.

**Table 5:** Association of human papillomavirus (HPV) DNA positivity with clinicopathological characteristics in non-small cell lung cancer (NSCLC) patients (*n* = 93)

Characteristics		HPV common				P value
		Negative		Positive		
		No	%	No	%	
Age	<=59	37	75.5	12	24.5	0.141
	> 59	27	61.4	17	38.6	
Gender	Male	44	68.8	20	31.3	0.983
	Female	20	69.0	9	31.0	
Smoking	Non-smoking	24	72.7	9	27.3	0.546
	Smoking	40	66.7	20	33.3	
Laterality (*n* = 77)	Left	22	71.0	9	29.0	0.895
	Right	32	69.6	14	30.4	
Histopathologic type	Adenocarcinoma	36	76.6	11	23.4	0.102
	Squamous cell carcinoma	28	60.9	18	39.1	
Stage (*n* = 90)	I & II	12	80.0	3	20.0	0.309
	III & IV	50	66.7	25	33.3	
Grade	1 & 2	41	70.7	17	29.3	0.616
	3	23	65.7	12	34.3	
Surgery	No	32	54.2	27	45.8	< 0.001
	Yes	32	94.1	2	5.9	
Metastatic	No	16	72.7	6	27.3	0.650
	Yes	48	67.6	23	32.4	

HPV: Human papillomavirus * Chi square or Fisher’s tests were used, p-values < 0.05 were considered significant

### Association between EGFR Mutations, HPV Infection, and clinico-pathological parameters in NSCLC patients

As shown in Table 6, EGFR mutations were identified in 19 of 93 NSCLC patients (20.4%), while the remaining 74 patients (79.5%) had wild-type EGFR. No statistically significant associations were observed between EGFR mutation status and age, gender, smoking status, histological subtype, tumor stage, grade, or HPV status (*p* > 0.05). Although EGFR mutations were slightly more common in patients aged over 59 years (22.7%) compared to those 59 and younger (18.4%) and among non-smokers (24.2%) than smokers (18.3%), these differences were not significant. EGFR mutations were more frequently observed in adenocarcinoma than SCC (27.7% vs. 13.0%), this difference was not statistically significant (*p* = 0.080). Similarly, a greater proportion of EGFR mutations was seen in lower-grade tumors (25.9% in grades 1&2 vs. 11.4% in grade 3; *p* = 0.094). Notably, a statistically significant association was observed between tumor laterality and EGFR status (*p* = 0.045), EGFR mutations more commonly detected in right-sided tumors (23.9%) compared to left-sided tumors (6.5%).

**Table 6:** Association of *epidermal growth factor receptor* (EGFR) status with various clinicopathological characteristics of non-small cell lung cancer (NSCLC) patients (*n* = 93)

Characteristics		EGFR				P value
		Wild		Mutant		
		No	%	No	%	
Age	<=59	40	81.6	9	18.4	0.603
	> 59	34	77.3	10	22.7	
Gender	Male	51	79.7	13	20.3	0.967
	Female	23	79.3	6	20.7	
Smoking	Non-smoking	25	75.8	8	24.2	0.499
	Smoking	49	81.7	11	18.3	
Laterality (*n* = 77)	Left	29	93.5	2	6.5	0.045
	Right	35	76.1	11	23.9	
Histopathologic type	Adenocarcinoma	34	72.3	13	27.7	0.080
	Squamous cell carcinoma	40	87.0	6	13.0	
Stage (*n* = 90)	I & II	11	73.3	4	26.7	0.564
	III & IV	60	80.0	15	20.0	
Grade	1 & 2	43	74.1	15	25.9	0.094
	3	31	88.6	4	11.4	
Surgery	No	48	81.4	11	18.6	0.574
	Yes	26	76.5	8	23.5	
Metastatic	No	18	81.8	4	18.2	0.765
	Yes	56	78.9	15	21.1	
HPV common	Negative	50	78.1	14	21.9	0.608
	Positive	24	82.8	5	17.2	

HPV: Human papillomavirus * Chi square or Fisher’s tests were used, p-values < 0.05 were considered significant

### EGFR mutations in NSCLC patients

**Fig. 1:**
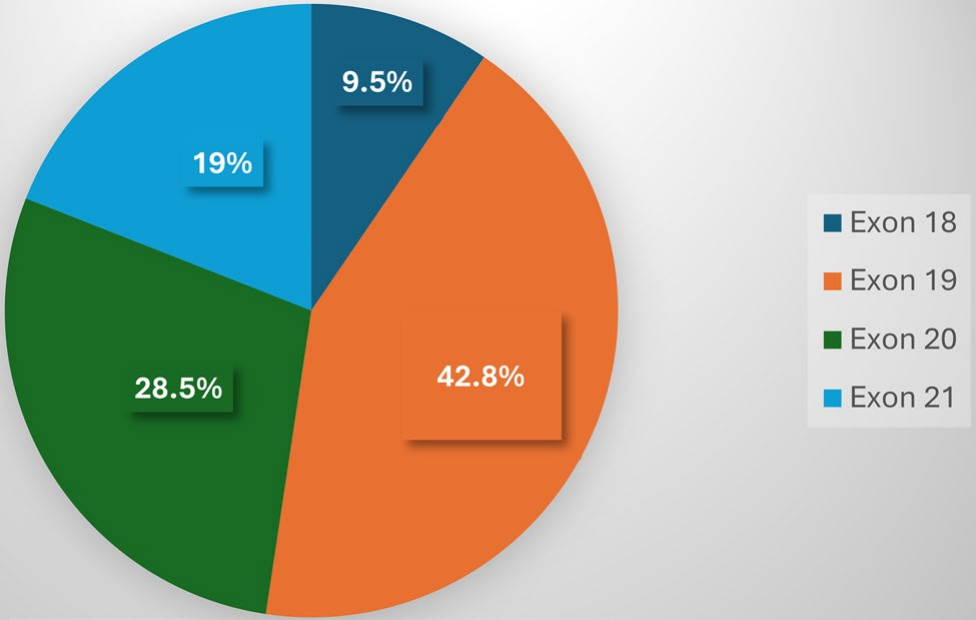
Distribution of *epidermal growth factor receptor* (EGFR) mutations by exon among EGFR-positive non-small cell lung cancer (NSCLC) patients (*n* = 19)

EGFR mutations were identified in 19 of 93 NSCLC patients, corresponding to a prevalence of 20.4%. A total of 21 mutations were detected: 17 patients harbored a single mutation, while 2 patients exhibited dual mutations. These mutations were distributed across four EGFR exons: exon 19 (42.8%), exon 20 (28.5%), exon 21 (19.0%), and exon 18 (9.5%) (Fig. 1). The most frequent alterations included: Exon 19 deletions (42.8%).

### Histologic subtype-specific distribution of EGFR mutations

**Fig. 2:**
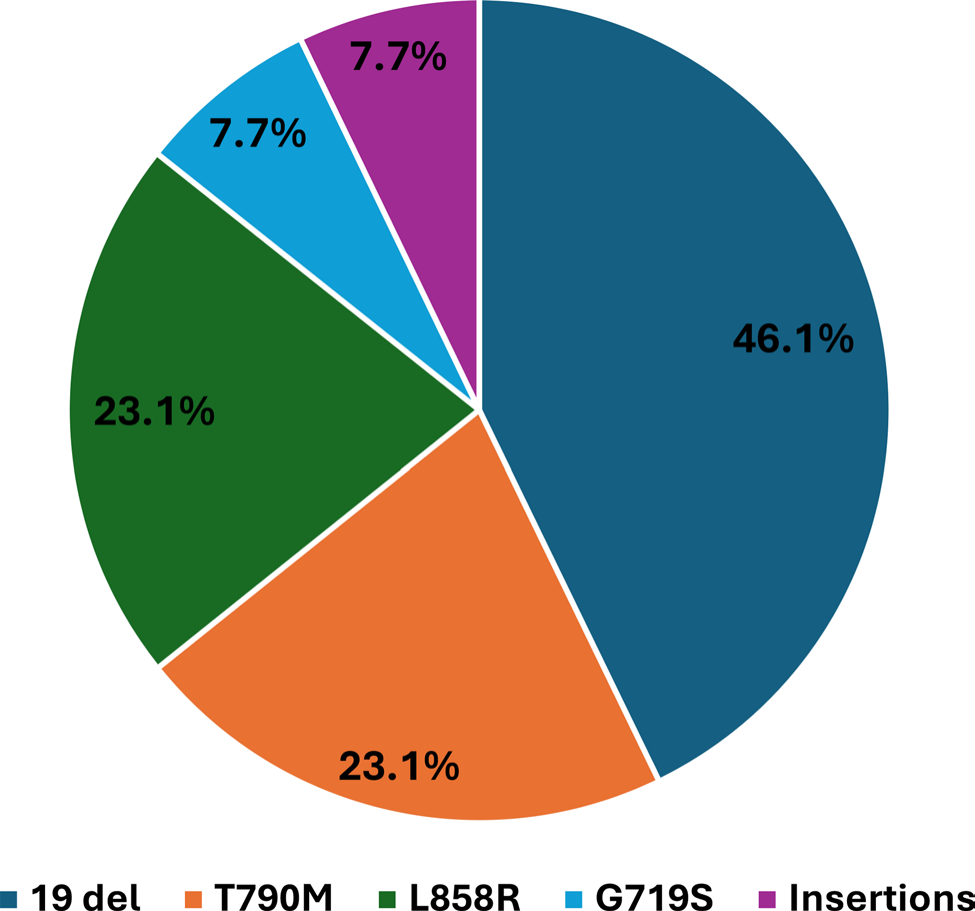
Distribution of *epidermal growth factor receptor* (EGFR) mutations among EGFR-positive adenocarcinoma patients (*n* = 13)

Among the 47 patients diagnosed with AC, 13 cases (27.6%) were positive for EGFR mutations. The most prevalent alteration was exon 19 deletions, observed in 46.1% of mutated AC cases, followed by L858R and T790M mutations, each present in 23.1% of patients. G719S (exon 18) and exon 20 insertion mutations were each detected in 7.7% of cases. Notably, one patient harbored dual mutations, a combination of an exon 19 deletion and T790M point mutation indicating the potential for complex resistance mechanisms even among tumors initially harboring sensitizing mutations (Fig. 2).

**Fig. 3:**
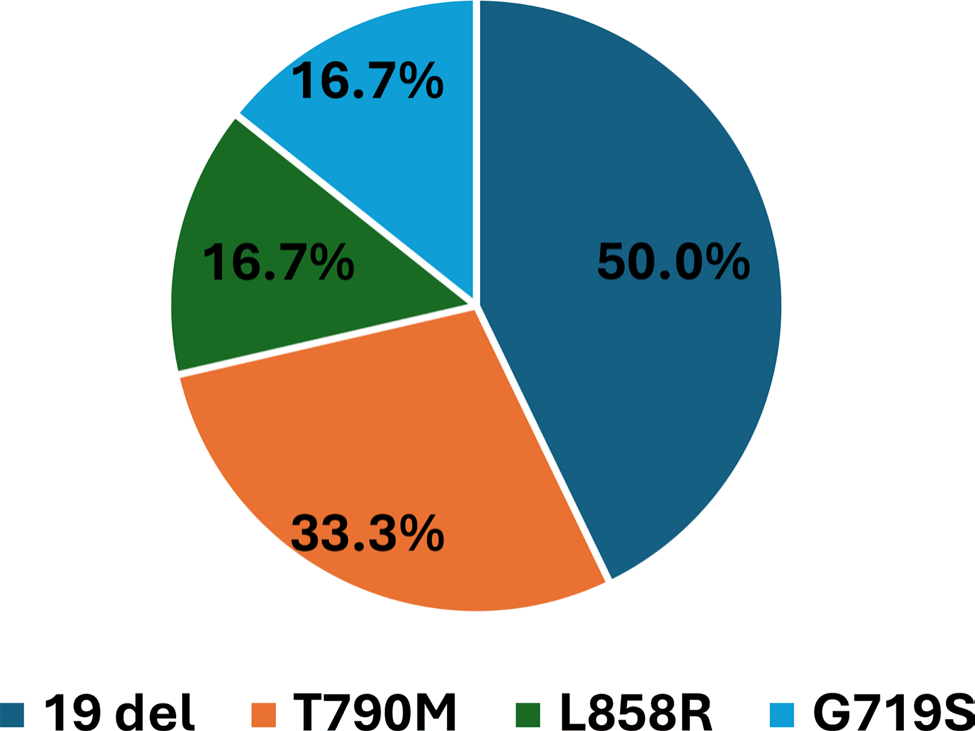
Distribution of *epidermal growth factor receptor* (EGFR) mutations among EGFR-positive squamous cell carcinoma patients (*n* = 6)

Figure 3 illustrates the EGFR mutations among SCC cases. In contrast to AC, EGFR mutations were significantly less frequent in SCC (13.0% (6/46) vs. 27.6% (13/47) in AC; (*p* = 0.136). Despite the lower overall prevalence, the mutation spectrum in SCC demonstrated considerable heterogeneity, with several clinically relevant alterations. Among the six mutation-positive SCC patients, the distribution of EGFR variants was as follows: Exon 19 deletions (50.0%), T790M (Exon 20; 33.3%), L858R (Exon 21; 16.7%), and G719S (Exon 18; 16.7%). Notably, one SCC patient harbored dual mutations (G719S + T790M). Given the small sample size, these subtype-specific frequencies should be interpreted descriptively rather than as statistically powered comparisons.

### Overall survival in NSCLC patients

**Fig. 4:**
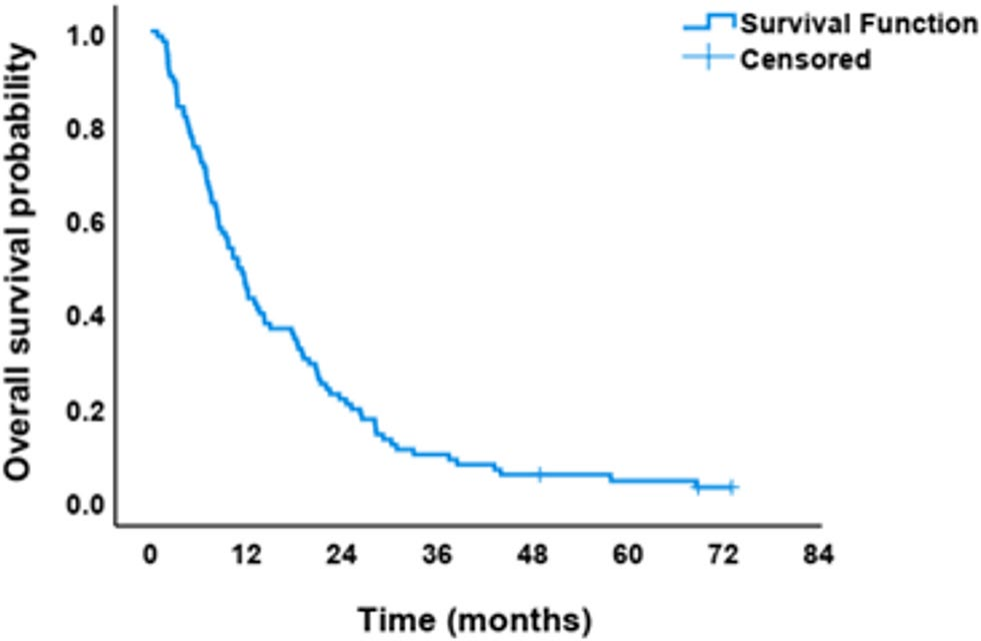
Overall survival curve for non-small cell lung cancer (NSCLC) patients (*n* = 93)

Figure 4 presents the OS curve for the cohort of NSCLC patients (*n* = 93). The curve illustrates the proportion of patients surviving over time following diagnosis or initiation of therapy. The survival curve demonstrates a gradual decline in NSCLC.

### Overall survival stratified by HPV status in NSCLC patients

**Fig. 5:**
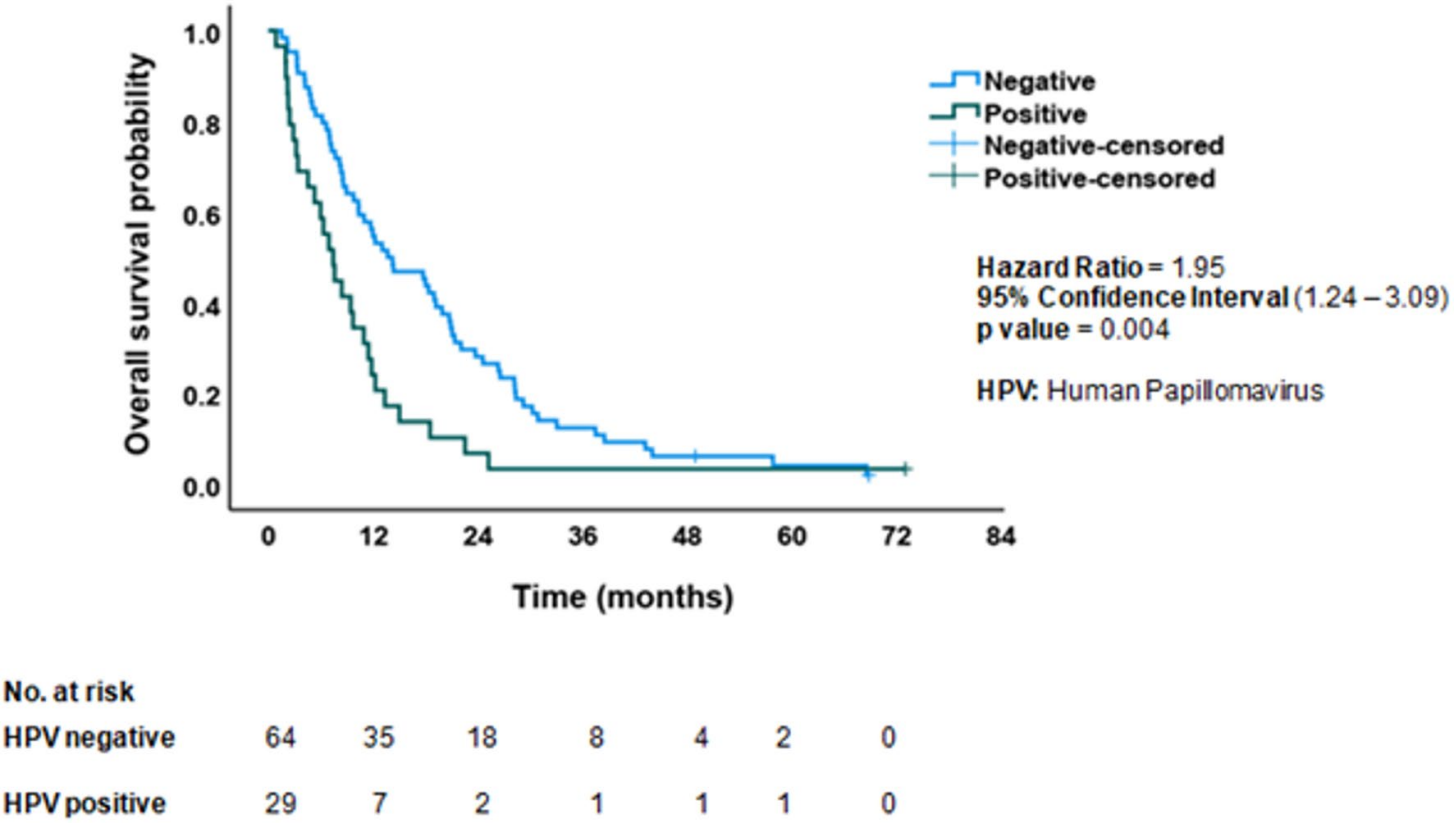
Overall survival curves stratified by human papillomavirus (HPV) status in non-small cell lung cancer (NSCLC) patients (*n* = 93)

Figure 5 presents a survival analysis of NSCLC patients (*n* = 93), stratified by HPV status (positive vs. negative for common high-risk genotypes). The figure compares survival outcomes based on the presence or absence of HPV DNA in tumor tissues. The HPV-positive group demonstrates an inclination toward shorter overall survival, potentially indicating an adverse prognostic influence of HPV infection. In contrast, the HPV-negative group appears to have relatively prolonged survival. The survival analysis emphasizes the potential prognostic significance of HPV infection in NSCLC. Kaplan–Meier analysis showed a significant difference in OS based on HPV status (*p* = 0.003).

### Overall survival based on combined HPV and EGFR mutation status in NSCLC patients

**Fig. 6:**
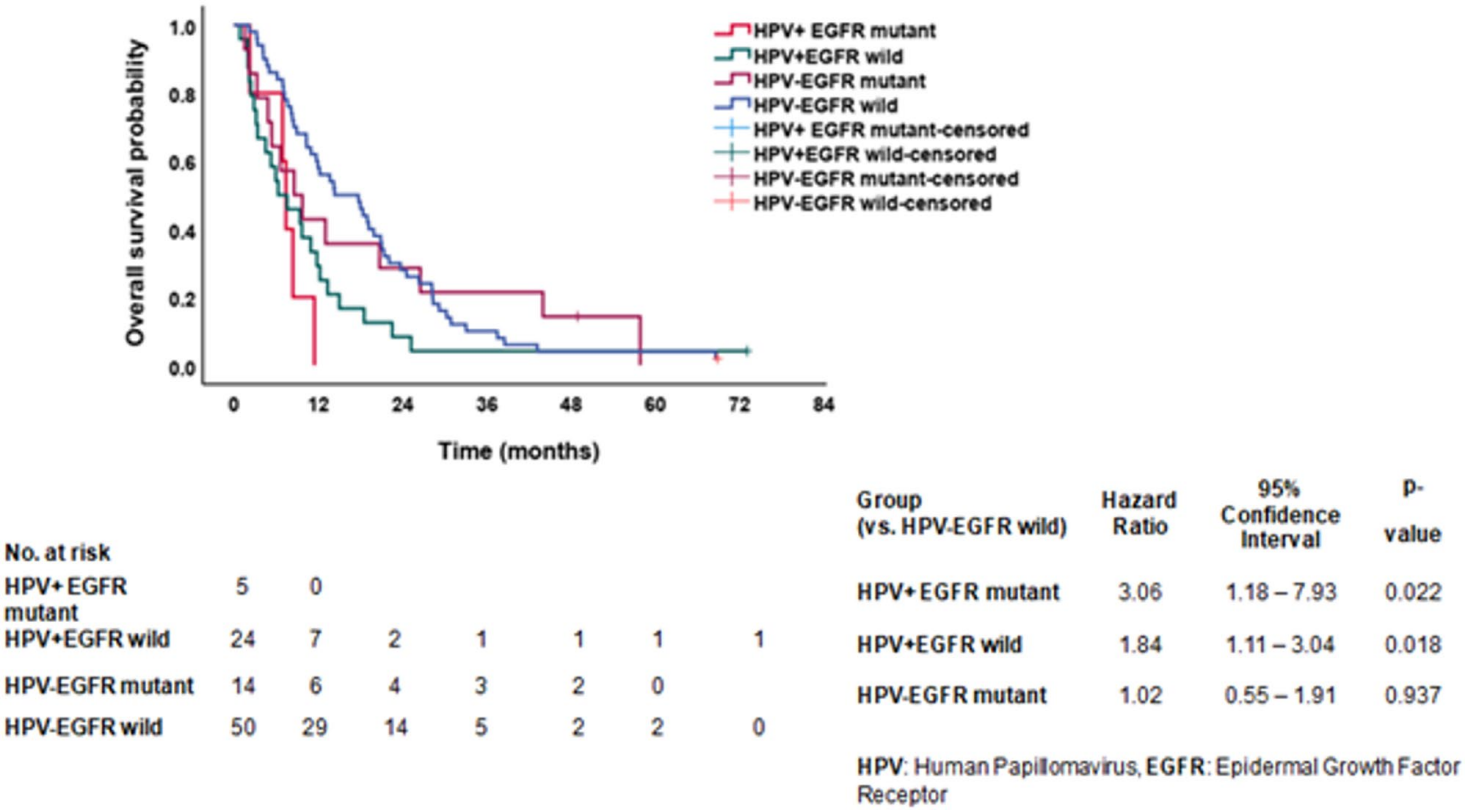
Overall survival curves stratified by combined human papillomavirus (HPV) and *epidermal growth factor receptor* (EGFR) mutation status in non-small cell lung cancer (NSCLC) patients (*n* = 93)

Figure 6 presents the OS curves for 93 NSCLC patients, stratified by combined HPV and EGFR mutation status. This analysis aims to evaluate the prognostic implications of viral oncogenesis (HPV infection) in conjunction with driver gene alterations (EGFR mutations).

Patients were categorized into four distinct molecular subgroups: HPV-negative/EGFR-wild type, HPV-negative/EGFR-mutant, HPV-positive/EGFR-wild type and HPV-positive/EGFR-mutant. EGFR-mutant groups, regardless of HPV status, tend to exhibit longer overall survival, likely reflecting the efficacy of EGFR-targeted therapies in this cohort. Patients with HPV-positive/EGFR-wild type tumors appear to have the shortest survival, potentially indicating a more aggressive clinical course and the absence of actionable mutations for targeted treatment. The subgroup with HPV-negative/EGFR-mutant status demonstrates the most favorable prognosis, suggesting a synergistic survival advantage in the absence of viral oncogenic influence and the presence of a targetable EGFR mutation. The analysis highlights the prognostic stratification value of integrating both viral and molecular profiling in NSCLC. Understanding how HPV infection interacts with EGFR mutational status can provide critical insight into patient risk stratification, therapeutic planning, and individualized treatment approaches.

Multivariate Cox proportional hazard analysis was conducted; the following variables were entered (which were significantly related to OS on univariate level); age, HPV& EGFR combination, laterality, stage, surgery and type of treatment, but HPV& EGFR combination wasn’t a significant predictor in this analysis.

## Discussion

Lung cancer remains the leading cause of cancer-related deaths worldwide, underscoring the need to elucidate its molecular and etiologic landscape. This study investigated the prevalence of HPV and EBV infections and their association with EGFR mutations in 93 Egyptian patients with NSCLC. The cohort was predominantly male (68.8%), with a median age of approximately 59 years, consistent with global NSCLC demographics [19]. The male-to-female ratio (≈ 2.2:1) indicates a rising proportion of female cases, mirroring global trends of increasing LC incidence among women, especially in developing countries [20].

Molecular profiling revealed that **EGFR mutations were present in 20.4% of cases**, with **exon 19 deletions and exon 21 L858R substitutions** as the most common variants—findings consistent with international data [21, 22]. The detection of **T790M mutations (exon 20)** in a subset of tumors supports their role in acquired resistance to earlier-generation EGFR tyrosine kinase inhibitors (TKIs) [23, 24]. Uncommon exon 18 mutations were also detected, known for their variable sensitivity to second-generation TKIs [25, 26]. These results emphasize the importance of comprehensive EGFR genotyping, ideally through next-generation sequencing (NGS), to ensure detection of both classical and atypical variants [27].

Virologic assessment revealed **HPV DNA in 31.2% of NSCLC cases**, with no detection in adjacent normal tissue, suggesting tumor-specific localization. HPV prevalence was higher in **squamous cell carcinoma (39.1%)** than in **adenocarcinoma (23.4%)**, consistent with the virus’s tropism for squamous epithelium [6]. **HPV-16** was the most frequent genotype (17.2% of HPV-positive cases), while other high-risk types (HPV 18, 33, and 58) were rarely detected [28, 29]. **EBV DNA** was found in only two cases, suggesting minimal contribution to typical NSCLC subtypes.

Clinically, HPV positivity was associated with lower resection rates, which may reflect a tendency toward more advanced disease presentation rather than a direct causal effect of the virus itself. No significant associations were observed between HPV and EGFR mutation status, implying potentially independent oncogenic pathways. Survival analysis showed a trend toward **poorer overall survival (OS)** among HPV-positive patients, whereas those with **EGFR-mutated/HPV-negative tumors** demonstrated better OS, consistent with favorable TKI responsiveness [21].

The distribution of **EGFR mutations** in this Egyptian cohort aligns with previously reported rates (15–25%) in Middle Eastern and North African population [30, 31], situated between the higher prevalence in East Asians and lower rates in Western populations. Similar to other studies, EGFR mutations were more frequent in **adenocarcinomas** and **non-smokers** [31, 32]. The predominance of exon 19 deletions and L858R mutations mirrors established global trends [33]. Detection of the **T790M resistance mutation** (23.8% of EGFR-mutant cases) reinforces its well-documented role in therapeutic resistance [23, 24].

When stratified by histology, the higher HPV prevalence in SCC parallels previous findings that associate HPV with squamous epithelial transformation [6, 34]. However, the detection of HPV-16 in adenocarcinoma diverges from common patterns observed in other HPV-driven cancers, suggesting potential tissue-specific viral interactions [28, 35, 36]. Global studies have reported HPV DNA in 10–30% of NSCLC cases, though with wide variability depending on region, detection method, and sample type [6, 37]. The high HPV prevalence in this Egyptian cohort may thus reflect regional differences or population-specific cofactors.

The lack of association between HPV and EGFR mutations is consistent with studies proposing **mutual exclusivity** between viral-driven and EGFR-driven oncogenesis [6, 34].

The detection of HPV DNA exclusively in tumor tissues raises the possibility of a **tumor-specific enrichment**, though not necessarily causal involvement. HPV’s known oncogenic mechanisms—E6- and E7-mediated inactivation of **p53** and **pRb**—have been implicated in several epithelial malignancies [38]. However, the role of these pathways in pulmonary tissue remains uncertain, particularly in the absence of functional evidence of viral activity.

The observed **correlation between HPV positivity and advanced disease** may reflect the combined effects of tobacco exposure, immune senescence, and impaired viral clearance in older patients [6]. This could facilitate viral persistence, chronic inflammation, and potential cooperation with other carcinogenic factors. Similarly, the predominance of right-lung tumors among EGFR-mutant cases, although not fully understood, may be related to anatomical or environmental factors influencing carcinogen exposure or airflow dynamics [39].

While EGFR mutations drive tumorigenesis through constitutive activation of downstream signaling pathways (e.g., PI3K/AKT, RAS/RAF/MEK), viral infections may modulate these same pathways, potentially enhancing tumor aggressiveness or altering therapeutic response [26, 40]. Nonetheless, the absence of a significant correlation between EGFR mutations and HPV infection in this study suggests these are likely **distinct molecular routes** to NSCLC development.

Although HPV detection in this study was based on PCR amplification of viral DNA, this approach has several important limitations that warrant consideration. PCR-based assays are highly sensitive for detecting viral DNA but cannot distinguish between transcriptionally active infections and incidental or latent viral presence. The detection of HPV DNA alone does not confirm oncogenic activity, as only transcriptionally active HPV marked by E6/E7 mRNA expression—is directly implicated in tumorigenesis [41, 42]. Moreover, PCR assays may yield false-negative results when viral integration disrupts the targeted genomic region (commonly L1), or when primer mismatches occur with variant or uncommon HPV types. Conversely, the high sensitivity of PCR renders it vulnerable to false positives due to contamination from laboratory reagents or adjacent HPV-infected tissues [43]. In addition, the use of qualitative rather than quantitative PCR limits assessment of viral load, which may influence clinical and biological relevance. To overcome these limitations, future studies should incorporate RNA-based assays or in situ hybridization to verify transcriptional activity, employ next-generation sequencing for broader HPV type coverage, and include rigorous contamination controls. Future studies should integrate multi-marker virologic testing (HPV DNA, RNA, p16 expression, and viral load quantification) with molecular and immunologic profiling to better elucidate the role of viral cofactors in NSCLC. Multi-center collaborations and prospective longitudinal studies are warranted to validate these findings, explore causal pathways, and assess potential clinical implications, such as viral screening or vaccine-based prevention strategies in high-burden regions.

## Conclusion

This study highlights the clinical relevance of high-risk HPV infection and EGFR mutations in Egyptian patients with NSCLC. EGFR mutations—particularly exon 19 deletions and L858R substitutions—were more frequent in adenocarcinomas and non-smokers and were associated with improved survival, especially in HPV-negative cases. Conversely, patients with HPV-positive/EGFR–wild-type tumors exhibited the poorest outcomes, suggesting a potentially high-risk subgroup that may require distinct therapeutic considerations. The observed inverse correlation between HPV positivity and surgical eligibility may reflect more advanced disease presentation rather than a direct viral effect.

While these findings underscore the prognostic value of integrating viral and molecular profiling in NSCLC, they should be interpreted with caution. The absence of p16 immunostaining and E6/E7 mRNA testing limits inference of viral oncogenic activity. Larger, multi-center studies incorporating functional viral assays are warranted to validate these observations and clarify whether HPV detection holds clinical or therapeutic relevance in lung cancer management.

## Data Availability

No datasets were generated or analysed during the current study.
